# Preview of partial stimulus information in search prioritizes features and conjunctions, not locations

**DOI:** 10.3758/s13414-019-01841-1

**Published:** 2019-09-03

**Authors:** Aave Hannus, Harold Bekkering, Frans W. Cornelissen

**Affiliations:** 1 Institute of Sport Sciences and Physiotherapy, University of Tartu, Tartu, Estonia; 2 Donders Institute for Brain, Cognition and Behaviour, Radboud University Nijmegen, Nijmegen, the Netherlands; 3 Laboratory for Experimental Ophthalmology, University Medical Center Groningen, University of Groningen, Groningen, the Netherlands

**Keywords:** Visual search, Conjunction processing, Color discrimination, Orientation discrimination, Feature equality, Feature preview, Precuing, Saccades

## Abstract

Visual search often requires combining information on distinct visual features such as color and orientation, but how the visual system does this is not fully understood. To better understand this, we showed observers a brief preview of part of a search stimulus—either its color or orientation—before they performed a conjunction search task. Our experimental questions were (1) whether observers would use such previews to prioritize either potential target locations or features, and (2) which neural mechanisms might underlie the observed effects. In two experiments, participants searched for a prespecified target in a display consisting of bar elements, each combining one of two possible colors and one of two possible orientations. Participants responded by making an eye movement to the selected bar. In our first experiment, we found that a preview consisting of colored bars with identical orientation improved saccadic target selection performance, while a preview of oriented gray bars substantially decreased performance. In a follow-up experiment, we found that previews consisting of discs of the same color as the bars (and thus without orientation information) hardly affected performance. Thus, performance improved only when the preview combined color and (noninformative) orientation information. Previews apparently result in a prioritization of features and conjunctions rather than of spatial locations (in the latter case, all previews should have had similar effects). Our results thus also indicate that search for, and prioritization of, combinations involve conjunctively tuned neural mechanisms. These probably reside at the level of the primary visual cortex.

## Introduction

Object recognition and visual search often require combining information on distinct visual features such as color and orientation. The question of which mechanisms and operations contribute to these visual processes has been at the heart of the work of Anne Treisman. Her classic contribution to this field—feature integration theory—proposed that during the search for combinations of such features, the features are first processed in independent modules, and only after then are they combined to represent more complex visual objects (Treisman & Gelade, 1980). The notion of features, and the questions of what constitutes a feature, and how features might be processed when in conjunction, fascinated Treisman (e.g., Treisman & Sato, 1990). The topic of features and their processing in conjunction has continued to interest researchers (Huang, 2015b; Wolfe & Utochkin, 2018). The present work links to these classic questions.

In a previous study, we found that in a conjunction search task, in which observers responded by making an eye movement to the selected item, the eyes were directed much more often to an item with the correct color than to one with the correct orientation (Hannus, van den Berg, Bekkering, Roerdink, & Cornelissen, 2006). Crucially, this was the case even though color and orientation selection efficacy had been fully equated based on prior single feature search experiments to achieve “feature equality” (Olds, Graham, & Jones, 2009). Comparable asymmetries have been described in other contexts: selection-for-action paradigms (Bekkering & Neggers, 2002; Hannus, Cornelissen, Lindemann, & Bekkering, 2005), in paradigms in which feature-cues were given prior to the conjunction search display (Anderson, Heinke, & Humphreys, 2010; Zhuang & Papathomas, 2011), in a feature preview paradigm (Olds & Fockler, 2004; Olds et al., 2009), and in double feature singleton search (Koene & Zhaoping, 2007; Zhaoping & May, 2007).

Those asymmetries suggest a dependency in the processing of basic features during conjunction search (Hannus et al., 2006; Zhaoping, May, & Koene, 2009). In this context, a dependency is when two conceptually distinct basic features are obligatorily processed together as a single entity. This may become apparent if the efficiency with which one basic feature (e.g., orientation) is processed depends on whether or not it is presented in combination with another basic feature (e.g., color).

The aim of the two experiments reported here was to understand how feature information is combined in search. We investigated this by using the approach known as temporal dissociation, precuing, or “feature previewing” (e.g., Allik, Toom, & Luuk, 2003; Olds et al., 2009; Sobel, Pickard, & Acklin, 2009). In this approach, partial stimulus information is presented prior to the remainder of that information. In our experiments, we presented a single feature of a conjunction and varied the amount of time before the complete conjunction stimulus was presented. For example, we revealed the color of the search items prior to the full color-orientation conjunction. The observer could complete the actual task—that is, decide on where the target is—only after the orientation information was added. Our research question was whether previewing would benefit performance. This knowledge could improve understanding of how processing dependencies influence search, which, in turn, could improve understanding of how information is combined to facilitate search and object identification.

In our analyses, we considered not only traditional performance in terms of correctly identified targets (hits) but also the correct selection of the target color or the target orientation (without this necessarily meaning that the exact target was found). We will refer to this as the feature selection efficacy (FSE) for color or orientation. We chose this approach because it is useful for analyzing performance and reasoning about underlying neural mechanisms (e.g., Hannus et al., 2006). Therefore, measuring FSE could help identify the strategy and mechanisms that observers used during search. To prevent floor or ceiling effects and to equate the FSEs for the different features (known as feature equality; Olds et al., 2009), we set them around threshold and determined them individually for each participant, on the basis feature search and prior to the main experiments (Hannus et al., 2006; and see the Method section). Consequently, we expected that previewing either orientation or color information would similarly affect performance.

In our experiments, we combined eye tracking and psychophysics. Using saccadic rather than manual responses has the advantage that these are fast and cannot be corrected once initiated. Saccadic responses can therefore provide a relatively direct view of the underlying mechanisms.

To derive more detailed predictions, we consider two possible strategies for using the previewed information in the subsequent search—namely (1) to prioritize potential target locations and (2) to prioritize features. These two strategies are not necessarily mutually exclusive, but, for the sake of simplicity, we treated them as such for now. For both strategies, we considered how a feature preview might affect performance and saccadic latency. Moreover, we assumed that performing the conjunction search task requires using either independent or dependent basic feature processing. As stated previously, dependent feature processing means that two conceptually distinct basic features are obligatorily processed together as a single entity.

## *Strategy 1: Preview prioritizes potential target locations*

In this strategy, the observer tries to enhance search by using the previewed information to separate potential targets (e.g., green items) from nontargets (red items). Subsequently, prioritization (or visual marking) of either target or nontarget locations may limit the effective set size for the search task (Kaptein, Theeuwes, & van der Heijden, 1995; Watson & Humphreys, 1997). For either independent or dependent basic feature processing, because of the reduced number of locations to process (reduced set size), relative to no previewing, we expected this strategy to result in improved performance and reduced latency of the saccadic response.

## *Strategy 2: Preview prioritizes features*

In this strategy, prioritization is conceptually similar to feature-based attention in that it globally enhances the processing of a specific feature (Boynton, 2005). In the case of independent basic feature processing, depending on the content of the preview, this strategy prioritizes one of the basic features that make up the actual conjunction search task. Because both features are ultimately required, this may be beneficial. Consequently, relative to no previewing, we may expect improved performance and reduced latency of the saccadic response.

In the case of dependent processing of features in conjunction search, using the feature previews for prioritization may be counterproductive, as only one of the features gets prioritized. Consequently, once the complete stimulus is shown in its entirety, this inappropriate feature will have to be deprioritized again. Hence, in this case, a preview may reduce performance and/or increase latency in the conjunction task. The preview is beneficial only if it contains information that matches sufficiently that of the complete stimulus. In that case, the preview may improve performance and/or reduce latency.

We performed two experiments, of which the second one elaborated on the first one and provided some additional control conditions. In both, we found that previews prioritize the processing of specific features rather than locations. The underlying process resembles reflexive, bottom-up feature-based attentional guidance, but also involves color and orientation-sensitive conjunction mechanisms.

## Experiment 1

In our first experiment, we previewed either the orientation or the color of the conjunction stimuli while manipulating the time between the onset of the preview and the onset of the complete conjunction (i.e., the preview duration).

### Method

#### Participants

Six volunteers (three females, between 22 and 32 years of age) participated in the experiment. All participants reported having normal or corrected-to-normal vision. Individual written informed consent was obtained. The study conformed to the research ethics guidelines of the University of Groningen.^1^

#### Apparatus

Stimuli were generated on a computer and presented on a 22-inch diameter CRT-monitor, driven at a resolution of 1,152 × 870 pixels and a refresh frequency of 75 Hz. The achromatic background luminance of the screen was 7.5 cd/m^2^. The distance between the eyes and the monitor screen was 50 cm. The software for experimental control was programmed in MATLAB using the Psychophysics and Eyelink Toolbox extensions (Brainard, 1997; Cornelissen, Peters, & Palmer, 2002; see http://psychtoolbox.org/).

Eye movements were recorded at 250 Hz with an infrared video-based eye tracker (EyeLink II; SR Research Ltd., Osgoode, Canada). Only the first saccade made upon complete stimulus presentation was analyzed. An eye movement was considered a saccade when the velocity of the eye was at least 25°/s, and the acceleration at least 9,500°/s, and the amplitude was at least 1°. The experiments took place in a closed, dark room. Participants rested their chin on a chin rest to prevent head movements.

#### Feature contrast determination

An important aspect in our experiments concerns the feature contrasts used to generate the conjunction stimuli. The “selectability” of the individual features should be equalized, otherwise it would be impossible to distinguish between biases resulting from contrast differences and those resulting from feature interactions. Therefore, prior to the main experiment, for each participant we first selected feature contrasts based on search performance during feature search. These individually equalized feature contrasts were then combined to define and generate the conjunction stimuli used in the actual experiment.

#### Procedure

Participants performed two single feature search tasks with different target/nontarget contrasts to determine individual thresholds for 70% discrimination accuracy for both color and orientation. Color contrasts were produced by increasing the luminance of one monitor gun (red or green) by a predetermined percentage (1.4%, 1.8%, 2.5%, 3.3%, 4.5%, 6.1%, 8.2%, 11.2%, 15.1%, or 20.4%) and decreasing the luminance of the other gun with the same percentage, so that total luminance stayed constant. Orientation contrasts were created by tilting the achromatic target and distractors in opposite directions relative to a baseline orientation of 45° and presenting them with an orientation difference of 1.8°, 2.5°, 3.3°, 4.5°, 8.1°, 11.2°, 22.4°, 30.2°, 40.8°, or 55.0°. Both tasks consisted of 260 trials (13 possible target positions × 10 contrast levels × one positive and one negative difference).

The 70% discrimination threshold value was interpolated after fitting a cumulative Gaussian function to the data.

#### Main experiment: Stimuli and procedure

**Fig. 1:**
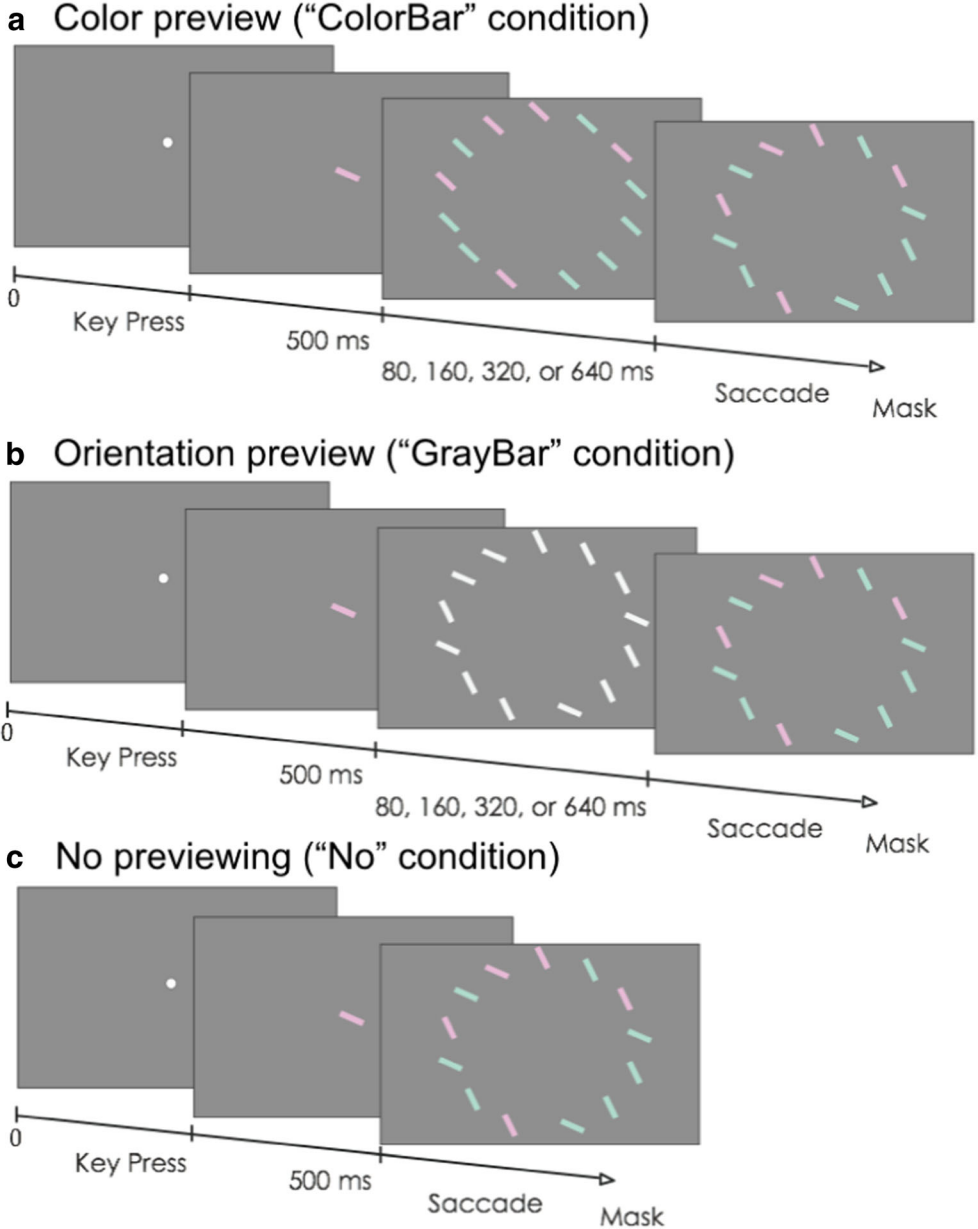
Precuing conditions in Experiment 1. For clarity, the bars in the three conditions have been drawn in exaggerated contrast. In the actual experiment, feature contrast were based on individually determined feature discrimination thresholds. (Color figure online)

Conjunction stimuli, of which the feature contrasts were adjusted based on these individually determined thresholds, were created from a factorial combination of two colors (green or red, where the contrast was based on the individual discrimination thresholds) with two orientations (clockwise or counterclockwise tilted, with a tilt difference relative to the baseline orientation of 45° based on the individual feature discrimination thresholds). The full conjunction stimulus consisted of 13 bars that were presented along the circumference of a circle with a diameter of 17 degrees that was centered on the fixation mark. Each bar was 3 × 0.5 degrees. Of the 13 bars, one bar was the target, four bars had the same orientation as the target but a different color, four bars had the same color but a different orientation, and the remaining four bars had both a different color and orientation from the target. Figure 1 schematically depicts the stimulus events in a trial. To start a trial, the participant fixated a central fixation mark and pressed the space bar. This was used also for drift correcting the eye-movement calibration. An example was then presented at the position of fixation mark to indicate which color and orientation the target would have in that specific trial. After 500 ms, the example disappeared, and the preview was presented. Two different preview conditions, and a third condition without a preview, were used. In the color-bar preview condition, all stimuli were uniformly oriented at 45°, with five stimuli having the target color and eight having the nontarget color. In the gray-bar preview condition, the stimuli were achromatic, with five having the target orientation, and eight having the nontarget orientation. Preview time was either 80 ms, 160 ms, 320 ms, or 640 ms,^2^ after which the preview was replaced by the full conjunction stimulus. In the no-preview condition, the conjunction stimulus presentation directly followed the target example. Upon detection of a saccade, the conjunction stimulus was replaced by a mask consisting of a large number of randomly oriented colored lines that covered the entire screen. Small black discs (<1°) were presented at each of the original bar locations to serve as saccade targets.

Participants were instructed to respond by making an overt eye movement to the perceived target location when the conjunction display was presented. In total, 468 trials were performed (13 target positions × two preview types × four targets × four preview times + the no-preview trials: 13 target positions × four targets). The trials had been randomized and divided into four blocks.

#### Analyses and statistics

All trials with saccades initiated within 100 ms after the presentation of the conjunction stimulus were removed because these were most likely express saccades (this procedure removed 2.3% of the data). Next, to evaluate overall search performance (hit rate), saccadic responses were classified into two categories:

1. Hit: The saccade was directed to the target (both color and orientation were correct).
2. Error: The saccade was directed to a nontarget.

The hit rate was calculated as the number of Category 1 trials as a proportion of the total number of trials (the sum of Category 1 and Category 2 trials). Hit latency was calculated as the average saccadic response time observed for the Category 1 trials. To evaluate FSE, saccadic responses were also classified into two additional categories:

A. Correct orientation selection: The saccade was directed to the target or a nontarget with the target orientation. Expressed as a percentage of the total number of trials, this is called the orientation selection efficacy (OSE).
B. Correct color selection: The saccade was directed to the target or a nontarget with the target color. Expressed as a percentage of the total number of trials, this is called the color selection efficacy (CSE).

To evaluate the presence of differences in hit rate, hit latency, OSE, and CSE as a function of preview type and preview time, we applied a series of repeated-measures analyses of variance (rmANOVAs) each with two within-subject factors: 2 preview types (color bar, gray bar) × 4 preview times (80 ms, 160 ms, 320 ms, 640 ms). To estimate the gains in FSE with respect to no previewing, we performed one-way rmANOVAs with five levels (0 ms, 80 ms, 160 ms, 320 ms, 640 ms). Scheffé’s method was used to control the overall confidence level in multiple comparison post hoc tests. Where necessary, the Greenhouse–Geisser correction was used to correct *p* values for violations of sphericity. A significance level of α = .05 was used for all statistical tests.

### Results

We observed pronounced differential effects of preview type and time on both the hit rate and CSE. A color preview tended to improve performance, whereas performance decreased following an orientation preview. We find that both hit latency and OSE were much less affected by preview type and time. These results are described in more detail below.

#### Overall search performance

**Fig. 2:**
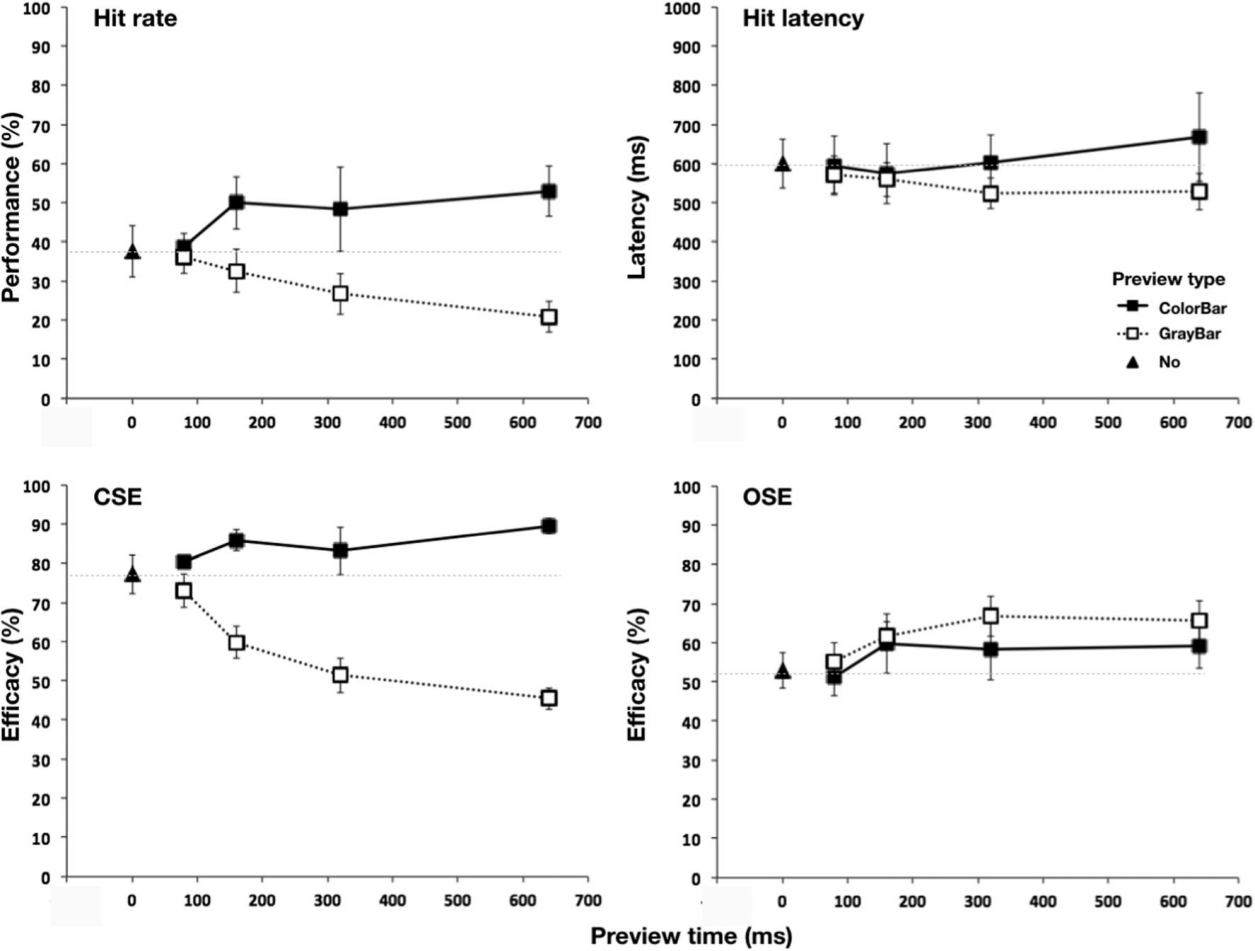
Effect of preview in Experiment 1. Panels show overall search performance (panel “hit rate”), saccadic response latency in case of hits (panel “hit latency”), color selection efficacy (panel “CSE”), and orientation selection efficacy (panel “OSE”). Error bars show standard error of the mean between observers. The dotted lines correspond to the no-preview level

Figure 2 (panel “hit rate”) shows search performance as a function of preview type and time. Preview type had a pronounced effect. Overall search performance was higher in the color-bar compared with the gray-bar preview condition. As a function of preview time, performance increased in the color-bar condition and declined in the gray-bar condition. These observations were confirmed by a main effect of preview type, *F*(1, 5) = 39.8, *p* = .001, η_p_^2^ = .89, and a clear Preview Type × Preview Time interaction, *F*(3, 15) = 6.4, *p* = .011, η_p_^2^ = .56. Planned comparisons between previewing type and the timing conditions revealed that performance following the color-bar and gray-bar previews started to deviate significantly from each other at 160 ms preview time and onwards—80 ms: *t*(4) = 0.8, *p* = .44; 160 ms: *t*(4) = 3.02, *p* = .030; 320 ms: *t*(4) = 3.3, *p* = .022; 640 ms: *t*(4) = 7.3, *p* < .001.

#### Saccadic latency

Figure 2 (panel “hit latency”) shows no clear effects of preview type and time. Statistics confirmed that neither preview type, *F*(1, 5) = 1.3, *p* = .31, η_p_^2^ = .21, nor preview time, *F*(3, 15) = 1.04, *p* = .36, η_p_^2^ = .17, had clear effects. No significant interaction was found between preview type and preview time, *F*(3, 15) = 4.4, *p* = .07, η_p_^2^ = .47.

#### Feature selection efficacy

Figure 2 (bottom panels) show CSE and OSE as a function of preview type and time. Baseline CSE (plotted at preview time zero) was approximately at the level expected based on the initial feature search experiments (76% on average; we expected 70%). In contrast, baseline OSE was markedly lower than expected (52% on average, we also expected 70%). This difference was significant, *F*(1, 5) = 18.0, *p* = .008, η_p_^2^ = .78.

#### Color selection efficacy

CSE strongly declined as a function of preview time in the gray-bar preview condition. In contrast, the one-way rmANOVA with five levels showed that the color-bar preview had only a marginal effect on CSE with no effect of preview time, *F*(4, 20) = 1.77, *p* = .21, η_p_^2^ = .26, and post hoc analyses showed no significant differences between the mean values of CSE (*p*s ≥ .25). For the gray-bar preview condition, however, we find a strong effect of preview time on CSE, *F*(4, 20) = 16.5, *p* = .001, η_p_^2^ = .77. Post hoc analyses showed that after 160 ms of gray-bar previewing, CSE was already significantly reduced compared with the no-baseline condition (*p* = .030). This effect became more pronounced with each sequential increase in preview time (*p*s < .01). The 2 × 4 factorial analysis confirmed a main effect of preview type, *F*(1, 5) = 148.5, *p* < .001, η_p_^2^ = .97, and showed a significant Preview Type × Preview Time interaction, *F*(3, 15) = 10.4, *p* = .002, η_p_^2^ = .68. Planned comparisons indicated that the difference between CSE in the color-bar and CSE in gray-bar conditions was not significant at 80 ms previewing, *t*(4) = 1.88, *p* =.119, but the increasing gap became significant at 160 ms previewing, *t*(4) = 5.29, *p* = .003, and longer, *t*(4) = 5.14, *p* = .004 for 320 ms, and *t*(4) = 16.26, *p* < .001 for 640 ms previewing.

#### Orientation selection efficacy

In both preview conditions, OSE changed similarly and relatively little. Indeed, a one-way rmANOVA showed no significant effect of preview time on OSE after color-bar pre-viewing, *F*(4, 20) = 1.7, *p* = .23, η_p_^2^ = .25 (post hoc comparison *p*s ≥ .43). The OSE plot suggested a trend towards higher OSE following longer gray-bar previewing, but this did not reach significance, *F*(4, 20) = 3.01, *p* = .07, η_p_^2^ = .38 (post hoc comparison *p*s ≥ .15). The 2 × 4 factorial analysis confirmed that there was no main effect of preview type, *F*(1, 5) = 2.6 *p* = .17, η_p_^2^ = .34, and no significant Preview Type × Preview Time interaction, *F*(3, 15) = 0.26, *p* = .79, η_p_^2^ = .049. Planned comparisons confirmed that the contrasts between the OSEs of any of the color-bar and gray-bar previewing timing conditions were marginal (*t*s ≤ 1.98).

### Discussion

Our main finding of Experiment 1 is a marked asymmetry in the effect of previewing either color or orientation information on subsequent visual conjunction search performance. Previewing color information slightly improved performance, whereas previewing orientation information reduced it. On the one hand, the improved conjunction search performance subsequent to a color preview (color bar) can be traced back to small improvements in both CSE and OSE. On the other, the reduced performance subsequent to an orientation preview (gray bar) can be traced back to a slight improvement in OSE combined with a strong reduction in CSE. No marked effects on saccadic latency were present.

We anticipated negative effects of a preview only when it would prioritize dependent feature processing in conjunction search (Strategy 2, dependent mechanisms). However, the results of Experiment 1 do not allow us to completely rule out a potential confounder. The previews of Experiment 1 may have also provided a general spatial cue, and not just prioritized features. Therefore, we conducted a control experiment (Experiment 2, described below) in which we introduced two additional previewing conditions.

## Experiment 2

**Fig. 3:**
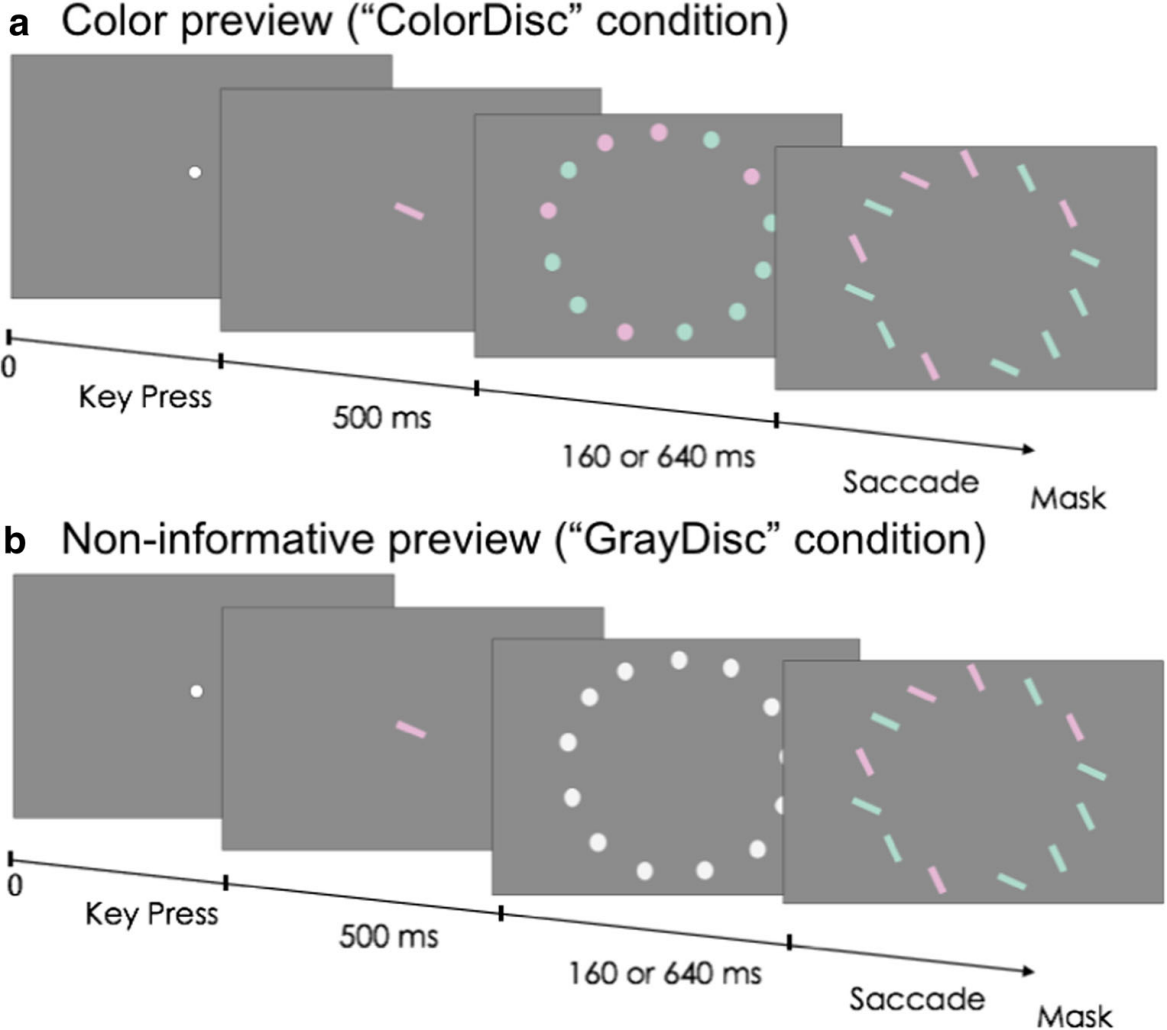
Additional disc precuing conditions in Experiment 2. For clarity, the discs and bars have been drawn in exaggerated contrast. For the actual experiment, feature contrasts were based on individually determined feature selection performance. (Color figure online)

In this second experiment, we compared the effect of four types of previews to the no-previewing condition. Besides the (a) color-bar and (b) gray-bar previews that were identical to the ones used in Experiment 1, we added two previews that contained no orientation information at all. The (c) color-disc preview consisted of discs of the same color as the color-bar preview (see Fig. 3a), whereas the (d) gray-disc preview consisted of achromatic discs of the same gray level as the gray-bar preview (see Fig. 3b). Therefore, this latter preview contained no task-relevant information at all and served to control for any aspecific temporal or spatial cueing effects.

### Method

#### Participants

Six participants (three females) with self-reported normal or corrected-to-normal vision participated in the experiment, of whom four had participated in Experiment 1.

#### Apparatus and stimulus materials

The paradigm was the same as that used in Experiment 1. The only differences were two additional preview types and a reduced number of preview times (160 ms, 640 ms).^3^ These additional previews consisted of discs with a surface size equal to that of the previews used in Experiment 1 and with either a luminance (gray disc) or color (color disc) identical to the gray-bar and color-bar previews, respectively. Participants performed 156 trials per preview type in two sessions. Each session consisted of four equally sized blocks of randomized trials.

#### Analyses

Principles of analysis and statistical procedures were similar to those applied in Experiment 1. To estimate the gains in overall hit performance or FSE with respect to no previewing, we performed one-way rmANOVAs with three levels (no ms, 160 ms, 640 ms). When testing the effects of preview shape and color, we performed rmANOVAs with within-observer factors preview color (color, gray), preview shape (bar, disc), and preview time (160 ms, 640 ms).

### Results

We reproduced the effects observed in Experiment 1 with the “bar” previews, whereas the “disc” previews had little to no influence on either performance or latency. These results are described in more detail below.

#### Overall search performance

**Fig. 4:**
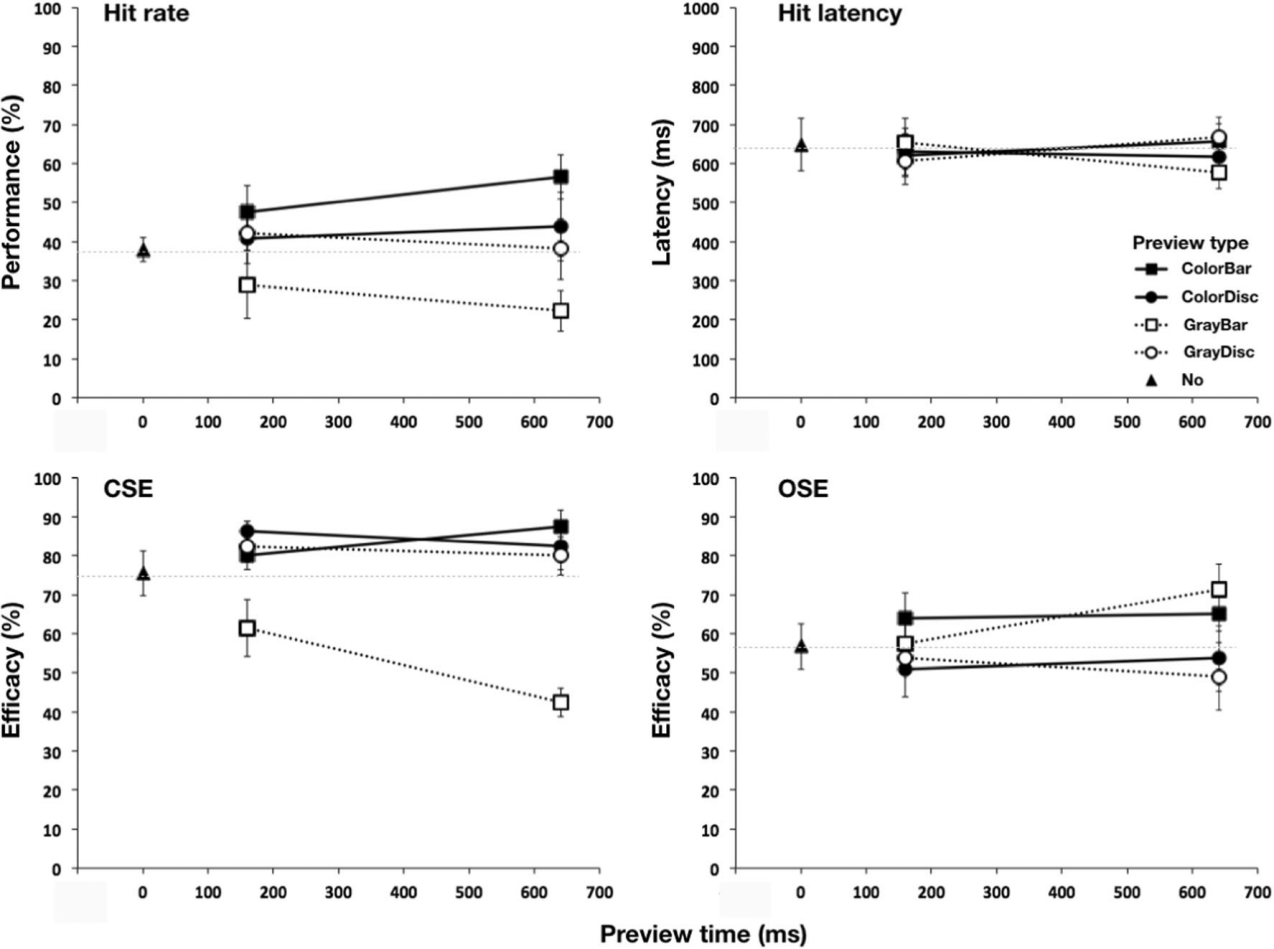
Effect of preview in Experiment 2. Panels show overall search performance (panel “hit rate”), saccadic response latency in case of hits (panel “hit latency”), color selection efficacy (panel “CSE”), and orientation selection efficacy (panel “OSE”). Error bars show standard error of the mean between observers. The dotted lines correspond to the no-preview level

Figure 4 (panel “hit rate”) shows search performance as a function of preview type and time. Again, our first observation was that preview type had a marked effect. Overall search performance was highest in the color-bar and lowest in the gray-bar preview condition. A series of one-way rmANOVAs with three levels indicated that in comparison to no previewing, search performance increased following a color-bar preview, *F*(2, 8) = 8.20, *p* = .027, η_p_^2^ = .67; this effect was explained by the significant performance increase from no preview to 640 ms preview (*p* = .012). Concurrently, there was a clear decreasing trend in the gray-bar condition, *F*(2, 8) = 5.27, *p* = .058, η_p_^2^ = .57, that could be attributed to the decline from no previewing to 640 ms previewing (*p* = .035). In both the disc conditions, such effects were absent (*F*s ≤ 0.83, η_p_^2^s ≤ .17). Further, the 2 × 2 × 2 rmANOVAs demonstrated a strong main effect for color, *F*(1, 4) = 110.0, *p* < .001, η_p_^2^ = .97, and a significant interaction between color and shape, *F*(1, 4) = 14.71, *p* = .019, η_p_^2^ = .79. Consequently, although search performance in general was much higher in the color than in the gray conditions, *t*(4) = 10.49, *p* < .001, for discs, the search performance was approximately equal in both the color and the gray conditions, *t*(4) = 0.63, *p* = .56, whereas for the bars, the search performance was significantly higher in the color than in the gray condition, *t*(4) = 7.75, *p* = .002.

#### Saccadic latency

Figure 4 (panel “hit latency”) indicates no systematic effect of preview type or time on saccadic latency—preview type, *F*(1, 4) = 0.36, *p* = .61, η_p_^2^ = .08; preview time, *F*(1, 4) < 0.1, *p* = .92, η_p_^2^ < .01.

#### Feature selection efficacy

Figure 4 (bottom panels) shows CSE and OSE as a function of preview type and time.

#### Color selection efficacy

As in Experiment 1, CSE declined following a gray-bar preview, *F*(2, 8) = 10.74, *p* = .006, η_p_^2^ = .73, whereas the two disc and color-bar previews had only small effects on CSE that did not reach statistical significance—color-bar: *F*(2, 8) = 3.3, *p* = .10, η_p_^2^ = .45; color-disc: *F*(2, 8) = 1.6, *p* = .27, η_p_^2^ = .29; gray-disc: *F*(2, 8) = 1.6, *p* = .27, η_p_^2^ = .29. We found a significant interaction between preview color and shape, *F*(1, 4) = 112.77 *p* < .001, η_p_^2^ = .97.

#### Orientation selection efficacy

As in Experiment 1, following both bar previews, OSE increased slightly (the increase from the no to the gray-bar conditions was significant), *F*(2, 8) = 9.27, *p* = .017, η_p_^2^ = .70, but the decrease from the no to the color-bar conditions was insignificant, *F*(2, 8) = 1.06, *p* = .39, η_p_^2^ = .21, which was different from the effects of both disc previews (confirmed by a significant main effect of preview shape), *F*(1, 4) = 16.00, *p* = .016, η_p_^2^ = .80.

### Discussion

First, Experiment 2 confirmed the main observations of Experiment 1. The gray-bar preview negatively affected performance and CSE, whereas the color-bar preview had a positive effect on performance. Secondly, the two disc previews had little to no effect on performance. Their effect on CSE resembled that of the color-bar preview, and so was markedly different from that of the gray-bar preview. Moreover, compared with the disc previews, the bar previews positively affected OSE. The differential effects of the disc and bar previews on performance and FSE support the notion that previews primarily caused prioritization of features, but not of spatial locations. In the latter case, all preview types, due to us ensuring “feature equality,” should have resulted in comparable changes in performance.

## General discussion

Our main finding is that the visual system uses previews of partial stimulus information to prioritize specific features rather than spatial locations. This conclusion is based on the following observations. Previews containing achromatic orientation information decreased performance on a subsequent color-orientation conjunction search task. Previews containing color information but no orientation information (discs) had little effect (comparable with a preview consisting of noninformative achromatic discs), whereas previews containing oriented color information enhanced overall performance (even though the orientation information itself did not inform about the task). Below, we discuss the implications of these findings and our conclusion in detail.

### Previews do not prioritize spatial locations

Our findings corroborate previous reports on an asymmetry in the effect of previews by Olds and Fockler (2004) and Olds et al. (2009) and extend those reports by showing that differential preview effects are also found when using saccade-based responses and with different stimuli. A potential confound could have been that observers searched only on the basis of the preview, and ignored the other feature relevant to the conjunction task. Since we established “feature equality” (Hannus et al., 2006; Olds et al., 2009), this is not a likely explanation for the asymmetry. Moreover, had observers searched primarily on the basis of the preview, we would have expected a pronounced reduction in latency, dependent on preview time, which we did not find.

In the Introduction, we presented two possible strategies for using previewed information: the prioritization of spatial locations or of features. We found compelling evidence against the notion that the preview was used for spatial prioritization (even though there was a strong incentive to do this, as the number of locations to search among could have gone down to five from the original 13). In both of our experiments, the oriented gray-bar and the oriented color-bar previews had differential effects on performance. Moreover, in our second experiment, the color-disc preview scarcely affected performance, whereas the oriented color-bar preview did. Yet, because of us ensuring feature equality, the gray-bar and the two color previews should have been similarly effective for spatial prioritization. Therefore, both of these findings run counter to the notion of spatial prioritization. In addition, saccadic latency remained stable irrespective of the preview time or visual performance, which also runs counter to the idea that prioritization had reduced the number of search locations (akin to the well-known set size effects in conjunction [serial] search). Visual marking (Watson & Humphreys, 1997) cannot explain our findings, either. It suppresses processing of existing items in displays to which new items are added. In our displays, all items were present already at preview, and all items changed from preview to conjunction task, which has been shown to abolish visual marking effects (Watson & Humphreys, 1997). In the case of spatial prioritization, a need to involve working memory (WM) could potentially underlie feature asymmetries. However, we deem this unlikely: not the feature information would have to be held in WM, but the location information, which is identical for all features. The differences in performance could also relate to the randomness of the preview displays (with the gray bar appearing more random and therefore less effective). However, this does not explain why a color-disc preview is less effective than a color-bar preview, even though both were similarly nonrandom. Because the previews do not appear to prioritize locations, the previews must have prioritized features and feature combinations. The latter assertion is supported below.

### Some features are more equal: A fingerprint of conjunctively tuned neural mechanisms in conjunction search and prioritization

The color-bar preview, containing both color and (noninformative) orientation information, may have acted as two overlapping previews that prioritized two independent features. In that case, the color should have slightly improved CSE (as we observed for the color-disc preview), while the orientation should have strongly reduced CSE (as we observed for the gray-bar preview). In combination, however, this should have resulted in a reduced CSE and not in the improvement that we observed for the color-bar preview. This implies that the color and orientation information of the previews were processed together as a single entity rather than independently. In turn, this implies that a conjunctively sensitive mechanism was responsible for the prioritization.

A conjoint representation of color and orientation in the visual system—known as the McCollough effect—was described 50 years ago (Houck & Hoffman, 1986; McCollough, 1965). Moreover, physiological studies have indicated that a considerable fraction of orientation-selective neurons in early visual cortex are also selective for color (Beaudot & Mullen, 2005; Hubel & Wiesel, 1968; Johnson, Hawken, & Shapley, 2008; Leventhal, Thompson, Liu, Zhou, & Ault, 1995; Livingstone & Hubel, 1984). On the basis of double singleton search tasks, mechanisms at the level of V1 and conjunctively sensitive to color and orientation have previously been implied in visual search (see also Koene & Zhaoping, 2007; Zhaoping & May, 2007). Our work corroborates this: Previews that contained both color and orientation information (color bar) apparently activated similar conjunctively sensitive neural mechanisms.

Other studies have shown that such color-sensitive orientation mechanisms have a somewhat coarser orientation tuning compared with their noncolor sensitive counterparts (Beaudot & Mullen, 2005; Webster, De Valois, & Switkes, 1990). Consequently, if such color-sensitive orientation mechanisms are used in performing a task, then orientation-selectivity should be somewhat reduced compared with that attainable during feature search. During conjunction search, OSE was indeed markedly lower than expected, whereas the CSE was approximately at the expected level. Hence, the results of both of our current experiments suggest the involvement of conjunctively sensitive mechanisms. Classic visual search theories, such as feature integration theory (Treisman & Gelade, 1980), hold that features are first processed independently and are then bound together at a later stage. Our study thus suggests that, at least for color and orientation, binding is early and occurs in neural mechanisms conjunctively tuned to color and orientation, which most likely reside at the level of the primary visual cortex.

Moreover, our results corroborate previous findings: “feature equality,” derived on the basis of feature search, does not imply equal feature selection efficacy during conjunction search (Hannus et al., 2005; Hannus et al., 2006; Olds & Fockler, 2004; Olds et al., 2009). Comparable asymmetries have been described in selection-for-action paradigms (Bekkering & Neggers, 2002; Hannus et al., 2005) and for conjunctions of orientation and size (Hannus et al., 2006). These asymmetries are thus a behavioral fingerprint (Zhaoping et al., 2009) of the involvement of conjunctively tuned neural mechanisms in search and preview. Our results also suggest that other asymmetries, such as differences in segmentation (Anderson et al., 2010), change detection (Huang, 2015a), grouping efficiency (Olds et al., 2009), and visual memory (Huang, 2015b), may ultimately derive from this asymmetric tuning of these conjunctive neural mechanisms in the color and orientation domains.

### A conceptual model of prioritization

**Fig. 5:**
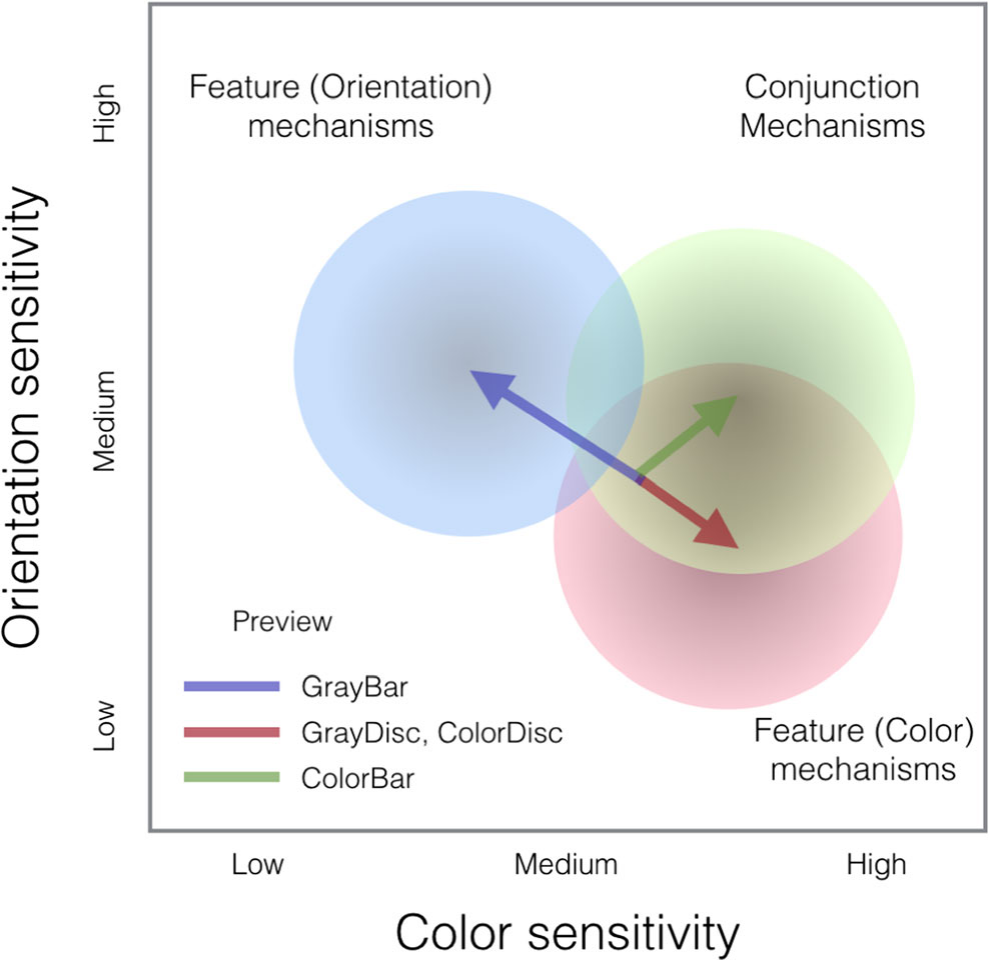
A conceptual model of prioritization in visual search. Depicted is a sensitivity space, in which putative neural mechanisms (i.e., neurons) are situated based on their sensitivity. Previews, depending on their content, cause prioritization by shifting the neural population’s sensitivity. (Color figure online)

Rather than prioritizing either color or orientation-sensitive neural mechanisms, the visual system appears to also prioritize a third mechanism that is conjunctively sensitive to color and orientation, resulting in at least a three-way prioritization process. Figure 5 depicts a conceptual model of the various previewing effects revealed by our present study. In the words of Treisman, when describing a model of perceptual processing, “think of it as a memory heuristic, a framework to give shape to the data currently available, rather than a fully-specified theory. It is certainly too simple and also certainly wrong in some respects” (Treisman, 1988, p. 203).

Figure 5 depicts a color and orientation sensitivity space, in which neural mechanisms (i.e., neurons) are situated based on their sensitivity. In the conventional view, prioritization would be restricted to feature mechanisms and follow the blue and red arrows. Our work indicates that this prioritization also engages conjunctively tuned mechanisms (green). Such conjunctively tuned mechanisms have a somewhat lower orientation sensitivity than achromatic oriented mechanisms, explaining asymmetries and dependencies in search and preview. Competition in search may thus arise between the chromatic (green) and achromatic (blue) orientation-sensitive mechanisms. Moreover, rather than being strictly three way, the prioritization is probably more continuous in reality and also involves other feature dimensions (e.g., spatial frequency).

An important question remains: How does prioritization affect search performance? Discriminating between items and selecting a target requires reading out the responses of neurons and subsequently comparing their responses. A preview provides time to optimize the selection of neurons for this read-out process, potentially increasing the signal-to-noise ratio and consequently improving search performance. Experiment 1 indicates that prioritization has its maximum effect after about 300 ms, which matches quite closely with estimates for guiding (feature-based) attention to color information (Lin, Hubert-Wallander, Murray, & Boynton, 2011; Palmer, Van Wert, Horowitz, & Wolfe, 2019). Hence, prioritization may also involve noise suppression and response tuning as found for feature-based attention (Boynton, 2005). In fact, it may be largely the same process, even though it should also comprise attending to “conjunction-based” mechanisms, as we have argued above. From a different perspective, a color-orientation conjunction, even though it can conceptually be decomposed into more basic features, is actually a feature to the visual system.

Prioritizing neurons for read-out on the basis of a preview will only be useful if the preview closely matches the stimulus. In our experiments, the color-bar preview, which contains both color and orientation information (although the orientation is noninformative), most closely matched the conjunction stimulus. Using this preview optimizes the set of neurons for read-out, thus improving search. In contrast, the gray-bar preview indicated that the selected neurons need only to be orientation sensitive and not color sensitive (which could be beneficial, as non-color-sensitive neurons tend to have higher orientation sensitivity). However, these neurons are suboptimal for the conjunction search task, resulting in reduced performance. That the gray bar was still used suggests that the prioritization is an obligatory, bottom-up process driven by the momentary visual information present in the scene, akin to what has been described for reflexive feature-based attention (Lin et al., 2011).

Still, an obligatory bottom-up process cannot explain all observations in the literature, as prioritization of search mechanisms appears also to be influenced by top-down processes such as action intention (i.e., whether to point or grasp; Bekkering & Neggers, 2002; Hannus et al., 2005) or the task at hand (e.g., the type of judgement to make; Jacobs & Cornelissen, 2017; Jacobs, Renken, Aleman, & Cornelissen, 2012; Jacobs, Renken, Thumfart, & Cornelissen, 2010). We nevertheless expect our results apply to both perception and action, as both appear to be based on the same underlying neural representations (Bruno, Knox, & de Grave, 2010; Eckstein, Beutter, Pham, Shimozaki, & Stone, 2007; Medendorp, de Brouwer, & Smeets, 2018; Yildirim & Cornelissen, 2015).

### Limitations and future directions

In the present experiments, specific choices were made regarding the approach and the features to be studied. In addition to hit rate and latency, we used OSE and CSE as outcome measures. Consequently, these needed to be in a range in which these could vary while avoiding floor and ceiling effects. As a result, we needed to generate stimuli using relatively weak feature contrasts, as visually healthy observers are extremely good in discriminating colors and orientation. In theory, we could have made feature contrast higher while retaining equality (e.g., taking a multiple of the thresholds), but this almost certainly would have made FSE (and in particular CSE) in conjunction search approach ceiling performance. It is possible that with higher feature contrasts, latency differences would have emerged that could reveal similar effects to those we observed here, but this remains to be determined. Conceptually, because of the global nature of feature-based attention with which prioritization shares many aspects, the previews may largely behave similarly to conventional feature cues. This also suggests that the actual locations of the previews may be irrelevant, which remains to be tested. While in the present study only color and orientation conjunction search was investigated, we would expect similar effects and asymmetries for other conjunctions such as color and size (Hannus et al., 2006), but not color and motion, for which no conjunctively tuned mechanisms appear to exist (Zhaoping et al., 2009). There is an interesting analogy in this to the concept of “integral vs. separable dimension pairs” in information visualization (Ware, 2013, p. ). Color-orientation pairs are among relatively integral dimensions. Size and orientation are considered even more integral. This suggests size and orientation conjunctions may show even stronger “feature inequality” in search and prioritization. Finally, our current conceptual explanation could be used to create a mathematical model based on population coding principles. Such a model could also license further predictions to be tested in future experiments.

#### Conclusions

The visual system uses previews of partial stimulus information to prioritize the processing of specific features rather than locations. This process is akin to reflexive, bottom-up feature-based attentional guidance, but comprises conjunctively tuned mechanisms.

## Footnotes

1 This study was part of a postgraduate research project conducted at the University of Groningen, and therefore conformed to the ethical guidelines of that institute.

2 In the experiment, preview times of 20 ms and 40 ms were also used. However, display timing verification after the experiments indicated that these preview times were not sufficiently accurate. Consequently, these trials were excluded from further analysis.

3 The second experiment also included trials with a 40-ms preview time. For the same reason as in Experiment 1, these were discarded.
